# Impact of negative energy balance and postpartum diseases during the transition period on oocyte quality and embryonic development in dairy cows

**DOI:** 10.3389/fvets.2023.1328700

**Published:** 2024-01-05

**Authors:** Idil Serbetci, L. Antonio González-Grajales, Carolina Herrera, Iulian Ibanescu, Muhittin Tekin, Manuel Melean, Fumie Magata, Eleni Malama, Heinrich Bollwein, Dragos Scarlet

**Affiliations:** ^1^Clinic of Reproductive Medicine, Vetsuisse Faculty, University of Zurich, Zurich, Switzerland; ^2^Department of Veterinary Medical Sciences, Graduate School of Agricultural and Life Sciences, The University of Tokyo, Tokyo, Japan; ^3^Institute of Veterinary Anatomy, Vetsuisse Faculty, University of Zurich, Zurich, Switzerland

**Keywords:** ketosis, endometritis, mastitis, oocyte/embryo quality, embryo kinetics

## Abstract

Transition period is a critical time for dairy cows because a large proportion of clinical and subclinical diseases are observed in the first month after parturition. Occurrence of negative energy balance is associated with depressed immunity and these conditions can affect oocyte quality and further embryonic development. The aim of this study was to assess the effects of negative energy balance-associated disorders on *in vitro* embryo production (IVP) in dairy cattle. We hypothesized that subclinical metabolic and/or inflammatory disorders have a negative effect on oocyte developmental competence and morphokinetic parameters of the resulting embryos. The study was conducted on 30 lactating Holstein-Friesian cows which were assigned into four groups: healthy (HEAL, *n* = 6), metabolic disease (META, *n* = 8), inflammatory disease (INFL, *n* = 8), or combined metabolic and inflammatory disease (COMB, *n* = 8). Ovum pick-up (OPU) was performed twice weekly on all cows over a period of four weeks (*n* = 8 OPU sessions/cow) starting on the fifth week postpartum, and the collected oocytes were subjected to routine IVP. Donor’s health status did not affect the number of oocytes/OPU or the recovery rate (*p* > 0.05). The number of quality 1 oocytes collected from INFL and COMB cows was lower compared to HEAL cows (*p* < 0.05). Also, the percentage of quality 1 embryos was reduced in META and COMB compared to HEAL cows (*p* < 0.05). Cleavage, blastocyst and hatching rates were similar among groups (*p* > 0.05). Presence of disease did not affect the time required by zygotes to reach specific developmental stages, as recorded by means of time-lapse monitoring. Nevertheless, there was a higher probability of direct cleavage after IVF in oocytes of COMB cows compared to those of HEAL cows (*p* < 0.05). In conclusion, oocytes and embryos derived from dairy cows diagnosed with subclinical metabolic and/or inflammatory diseases during the transition period showed reduced quality but similar developmental potential and morphokinetics when compared to healthy cows. These results shed light on the consequences of subclinical disease on embryonic development in dairy cows which might be important for embryo transfer programs.

## 1 Introduction

The transition period is a crucial phase for dairy cows as up to 75% of clinical and subclinical diseases are observed in the first month after parturition (1, 2). During this period, cows exhibit a reduced proliferation of peripheral blood mononuclear cells, an indicator of cell-mediated immune function (3). Moreover, cows experience severe negative energy balance at the onset of lactation due to increased nutritional requirements for milk production and maintenance exceeding the dry matter intake (4). As a consequence, the concentration of non-esterified fatty acids (NEFA), B-hydroxybutyrate acid (BHBA), urea, and ketone bodies in the blood increases (5, 6). This can lead to subclinical ketosis, which is prevalent in 24.3% of dairy cows worldwide (7). Remarkably, these changes are not only observed in the blood but also in the follicular fluid of dominant follicles (8, 9).

A healthy intrafollicular environment is essential for the acquisition of developmental competence by the oocyte (10). Due to negative energy balance, the oocyte is exposed to high NEFA concentrations during the final stages of maturation (11). Saturated NEFA exert a toxic effect on the oocyte (12), but this can be in part counteracted by the cumulus cells (13). Elevated NEFA concentrations were shown not only to reduce oocyte developmental competence, but also to affect embryo quality, viability and metabolism (14). Another typical manifestation of the imbalance between dry matter intake and the requirements for milk yield in dairy cows is low blood glucose concentration (15, 16). Glucose is an indispensable molecule for metabolic processes during oocyte maturation (17) and cumulus expansion (18). It has been reported that reduced blood glucose concentration might impair cleavage and subsequent blastocyst development (19, 20). Conversely, BHBA can also exert further toxic effects on the oocyte and the developing embryo but only under hypoglycaemic conditions (19). On the other hand, embryos from cows with high BHBA seem capable to tolerate dietary energy deficits and become more energy efficient (21). Nevertheless, as most of the abovementioned studies only mimicked specific conditions *in vitro*, it is not still clear to which extent (sub)clinical ketosis would affect embryonic development in an *in vitro* embryo production (IVP) program.

Besides its direct effects on embryonic development, ketosis was also shown to reduce immunoresponsiveness by impairing neutrophil and lymphocyte function (22, 23). In turn, the incidence of inflammatory diseases in dairy cows increases to a prevalence as high as 20%–50% for mastitis, 40% for metritis, and 20% for endometritis (24–26). All these conditions exert deleterious effects on fertility due to cytokine release into circulation, thus affecting ovarian and uterine function (27, 28). Cows with uterine inflammation show slow follicular growth and lower steroid hormone concentration, consequently delaying the resumption of ovarian activity (29, 30). Moreover, the capacity of the oocytes from endometritis cows to develop to morulae stage after *in vitro* fertilization (IVF) is reduced (31). Similarly, mastitis cows present impaired follicular growth and steroidogenesis (32, 33), but also reduced oocyte quality and subsequent embryonic development (34), most likely due to the presence of pro-inflammatory cytokines also in the follicular fluid. In transition cows, the individual degree of inflammation can reliably be determined as haptoglobin mirrors and significantly positively correlates to both body temperature and Metricheck score (35).

Identifying viable embryos with high developmental potential after transfer to a recipient is essential for improving success of IVP programs (36). Conventionally, the quality of *in vitro* produced embryos is evaluated based on their morphology, but this criteria is regarded as subjective and therefore not optimal (37, 38). Time-lapse monitoring (TLM) is an alternative, non-invasive method which allows the analysis of morphokinetic parameters such as timing of cell division and blastomere number in individual embryos (39–41). Several studies showed that the time required until first cleavage is linked to the implantation potential of the embryo (42, 43). Additionally, viable embryos that develop to the compact morula or blastocyst stage have shorter first and second cell division cycle than non-viable embryos (44).

Clinical metabolic and/or inflammatory diseases impair reproductive performance in dairy cows, but the extent to which cows with subclinical disease can be enrolled in a commercial IVP program remains unclear. The aim of this study was to assess the effect of subclinical inflammatory and/or metabolic disease during the transition period on the number, quality, developmental competence as well as morphokinetic characteristics of oocytes collected by ovum pick-up and undergoing *in vitro* fertilization. We hypothesized that the number and quality of oocytes is reduced in cows suffering from subclinical ketosis and/or inflammatory disease and this leads to reduced developmental competence and altered cleavage patterns of the resulting embryos as assessed by time-lapse monitoring.

## 2 Material and methods

All experimental procedures have been carried out in accordance with the Swiss legislation for animal welfare and were approved by the Committee on Animal Experimentation of the Cantonal Veterinary Office Zurich (license no. 131/2018).

### 2.1 Animals

The study was conducted on 30 lactating Holstein-Friesian cows from July 2019 to June 2021 at AgroVet-Strickhof, Lindau, Switzerland. One cow was included in the study in two consecutive lactations. All cows were housed in a free-stall barn with a gummy bedded pen for the duration of the experiment and fed a total mixed ratio composed of maize and grass silage with concentrate based on milk yield. The water was provided *ad libitum* throughout the study. At the start of the experiment, the average age of the animals was 55.0 ± 17.6 months and the parity was 2.4 ± 1.3. The average milk yield adjusted to 305 days of lactation per cow was 10,865 ± 1,126 kg.

Cows underwent clinical examination 60, 30, and seven days before the expected calving date, within 24 h postpartum, as well as once per week during the first 60 days after calving to detect signs of metabolic and/or inflammatory disease. Rectal temperature, heart rate, respiratory rate, and rumen filling were monitored (45) and physical examination of the udder was performed. At each clinical examination, body condition score (BCS) and back fat thickness (BFT) were also recorded. Body condition score was assessed based on a scale of 1 to 5, with increments of 0.25, where BCS 1 reflected emaciated and BCS 5 reflected obese animals, as previously described (46). Back fat thickness was recorded using ultrasonography as recommended (47). Cows with conditions such as twins, stillbirth, uterine torsion, ovarian cysts, lameness were excluded from further sample collection for the study.

### 2.2 Gynecological examination

Gynecological examination was performed weekly during the first month after calving. The reproductive tract was palpated transrectally and uterine location, horn symmetry, uterine fluid, and contractility were assessed. After palpation, the ovaries and uteri of all cows were scanned with a transrectal ultrasound scanner (MyLabOne VET, Esaote, Genoa, Italy) equipped with a multi-frequency 5- to 10-MHz linear array transducer to assess ovarian activity and presence of intraluminal fluid in the uterus. Afterwards, vaginoscopy was performed to observe vaginal mucosa, cervix and, in case discharge was present, it was qualitatively scored as clear, predominantly clear with some flecks of pus, mucopurulent or purulent, respectively.

### 2.3 Blood analyses

At each clinical examination, 10 mL blood was collected from the coccygeal vein into vacuum tubes with and without anticoagulant (Vacuette, Greiner Bio-One, Kremsmünster, Austria), respectively. Within 5 min after sampling, concentration of B-hydroxybutyrate acid (BHBA) was determined with an electronic hand-held measuring system (BHB-Check, TaiDoc Technology Corporation, Taiwan). The results were expressed as mmoL/L and the BHBA threshold for subclinical ketosis was 1.2 to 2.9 mmoL/L, as previously described (48). Afterwards, serum and plasma underwent centrifugation (2000 × g, 15 min at 4°C) and were stored at −20°C until further analysis. A commercial ELISA kit (OKIA00002, Aviva Systems Biology, San Diego, CA, United States) was used to determine haptoglobin concentration, as an indicator of inflammation, according to the manufacturer’s instructions. Intra- and interassay CV was 6.0% and 5.9%, respectively. The minimum detection limit of the assay was 15.6 ng/mL.

### 2.4 Milk analyses

From week 2 to week 8 after calving, milk quality was analyzed weekly. Initially, California Mastitis Test (CMT) was performed for individual udder quarters to screen for mastitis. The CMT results were scored as 0 (negative), 1 (weak positive), 2 (distinct positive), and 3 (strong positive) based on thickening or gel formation (49). Cows were considered positive for mastitis if CMT score > 0 was observed in at least one of the quarters. Furthermore, 10 mL milk from each quarter was collected and mixed in a sterile 50 mL Falcon tube to determine somatic cell count (DeLaval, Hamilton, New Zealand). Mastitis was diagnosed if the somatic cell count was >200.000 cells/mL (50).

### 2.5 Group assignment of the cows according to their health status

Based on the results of clinical and laboratory analyses, cows were retrospectively assigned to one of the following groups: healthy (HEAL, *n* = 6 cows, free of any signs of metabolic and/or inflammatory disease), with metabolic disease (META, *n* = 8 cows, serum BHBA 1.2 to 2.9 mmoL/L at week 5 and 8 postpartum), with inflammatory disease (INFL, *n* = 8 cows, somatic cell count >200.000 cells/mL and/or vaginal discharge and plasma haptoglobin >15.6 ng/mL at week 5 and 8 postpartum), and with a combination of metabolic and inflammatory disease (COMB, *n* = 8 cows, concurrent signs of metabolic and inflammatory disease at week 5 and 8 postpartum).

### 2.6 Ovum pick-up

Ovum pick-up (OPU) was performed twice weekly (Monday and Thursday) on all cows over a period of four weeks (*n* = 8 OPU sessions/cow) starting on week 5 postpartum. Three different operators were arbitrary assigned to perform the OPU procedures. An ultrasound scanner (ExaPAD mini, IMV Imaging, Angoulême, France) equipped with a 6.5 MHz transvaginal ultrasound probe together with 18G needles connected to an aspiration pump (MOFA Global, Verona, WI, United States) were used for follicular aspiration. Cows received epidural anesthesia (5–6 mL lidocaine, Lidocain 2%, G. Streuli & Co. AG, Uznach, Switzerland) 5 min prior to OPU. Then, the number of follicles on each ovary was recorded, and all visible follicles (≥ 3 mm) were aspirated. The fluid was collected into 50 mL tubes containing phosphate-buffered saline supplemented with 5 IU/mL heparin and held at 38°C on a heat block. Cumulus-oocyte complexes (COCs) were identified under a stereomicroscope and transferred to 35 × 100 mm petri dishes (Corning, New York, United States) containing BO-Wash medium (IVF Bioscience, Cornwall, United Kingdom). Oocyte quality was then assessed by a single person based on morphology of cumulus cells and ooplasm homogenity and graded as follows: grade 1, more than four layers of compact cumulus cells with homogenous ooplasm, grade 2, with one to three layers of cumulus cells and homogenous ooplasm; grade 3, denuded oocytes and irregular ooplasm; grade 4, oocytes with expanded cumulus cells and irregular ooplasm with dark clusters. Grading was done according to criteria published in the 5th Edition of International Embryo Technology Society (IETS) Manual (accessed November 2023). Cumulus-oocyte complexes retrieved during the first OPU session of each week (*n* = 4 OPU sessions in total) were used for IVP, whereas COCs recovered during the second OPU session of each week were stored for further analyses.

### 2.7 *In vitro* embryo production

From the total of 1,424 collected COCs, 163 in HEAL, 226 in META, 201 in INFL and 216 in COMB underwent *in vitro* maturation (IVM) in pre-equilibrated 5 mL tubes containing BO-IVM^®^ media (IVF Bioscience) covered with mineral oil (Sigma-Aldrich, Steinheim, Germany) at 38.2°C, 5% CO_2_ in air and saturated humidity for 20–22 h. Frozen semen from the same ejaculate of the same Fleckvieh bull with known *in vitro* fertility was used for all IVF runs. After thawing, semen was placed on top of 750 μL Percoll 90% and centrifuged for 15 min at 600 × g. The pellet was resuspended in 750 μL of Hepes-buffered Tyrode’s albumin lactate pyruvate medium and centrifuged for 3 min at 600 × g. Then, the pellet was transferred to pre-equilibrated BO-IVF^®^ fertilization medium (IVF Bioscience), and the sperm concentration was calculated using a hemocytometer (Neubauer chamber). Each IVF droplet contained up to 20 COCs inseminated with 1 × 10^6^ sperm/mL and the day of IVF was considered Day 0. Co-incubation of COCs with sperm took place at 38.2°C, 5% CO_2_ in air and saturated humidity. Twenty hours after IVF, presumptive zygotes were stripped from their cumulus cells using a 135 μm stripper pipette (CooperSurgical, Inc., Trumbull, CT, United States) and then transferred in groups of 10 to BO-IVC^®^ culture medium (IVF Bioscience), covered with mineral oil, and *in vitro* cultured (IVC) at 38.2°C, 5% CO_2_, 5% O_2_, and saturated humidity. Fourty eight hours after IVF (Day 2), non-cleaved oocytes were separated and placed in a different culture droplet, while cleaved embryos were further cultured until Day 9. Blastocyst development was recorded on Day 7, 8, and 9 days after fertilization. One embryo was considered hatched if it has emerged from the zona pellucida completely. The quality of the embryos was assessed as excellent (grade 1), fair (grade 2), poor (grade 3) or degenerated (grade 4), according to the recommendations of the 5th IETS Manual. Only the hatched embryos were considered for statistical analysis of embryo quality.

### 2.8 Time-lapse monitoring

A total number of 246 presumptive zygotes (HEAL: *n* = 46; META: *n* = 83; INFL: *n* = 60; COMB: *n* = 60) from OPU sessions 4 and 8 from each cow were monitored using the PrimoVision TLM (Vitrolife, Göteborg, Sweden) placed inside the incubator. The presumptive zygotes (3–18 zygotes/cow) were cultured in a 9- or 16-microwell culture dish (Vitrolife) and were imaged every 10 min from Day 1 to Day 9 of development by TLM to record the following: time of first cleavage (t1), time of second cleavage (t2), time of last cleavage before entering the lag phase (t3), duration of the lag phase (dLP), time of cleavage resumption after lag phase (tRCl), starting time of blastocyst expansion (tSB), starting time of hatching (tHB) and ending time of hatching (tHDB), respectively. Furthermore, the patterns of first cell division were evaluated and abnormal cleavage, i.e., direct or reverse cleavage, recorded as previously described (41).

### 2.9 Statistical analysis

A non-parametric Kruskal Wallis test followed by a Bonferroni multiple comparison *post hoc* test was performed to test for differences regarding serum BHBA and haptoglobin concentration and time of cell division among groups. A two-way mixed ANOVA was performed to evaluate the effects of health status (group), the week postpartum and the interaction group × week on BCS and BFT score. Pairwise comparisons were run between different time points (postpartum weeks) for each group, with a Bonferroni adjustment applied to control the family-wise error rate. The effects of the progressing number of OPU, the operator conducting the OPU and the health status of the donor on follicle number, oocyte and embryo number and recovery rate were assessed by fitting mixed-effects linear models with a first-order autocorrelation structure. The interaction terms operator × group and group × OPU were also included as fixed effects, while the effect of the cow was considered as a random one. A Bonferroni chi-square residual analysis was performed to test for differences regarding recovery rate, oocyte quality, cleavage rate, blastocyst rate, hatching rate, and embryo quality among groups. To assess group-related differences in the incidence of direct or reverse cleavage, contingency tables 2×4 (dichotomous outcome for direct or reverse cleavage by four health status groups) were created to present the absolute as well as the % relative count of the two variables per group. Generalized linear models were separately fit to model the binary response variables “direct cleavage” and “reverse cleavage” (0 = no occurrence, 1 = occurrence) as a linear function of the explanatory variable ‘health status group’. The logit function was used to link the response variables to the linear model. The alternative hypothesis (predictor coefficients in the regression equation differ from zero) was tested against the null hypothesis at *p* = 0.05 significance level. Binary logistic regression was performed for embryos that reached the blastocyst stage. Posthoc pairwise comparisons between the four health status groups were performed using Z-tests corrected with Holm’s sequential Bonferroni procedure. Data are presented as mean ± SD. A difference was considered significant when *p* < 0.05.

## 3 Results

### 3.1 Blood analyses and parity of the cows

**Figure 1:**
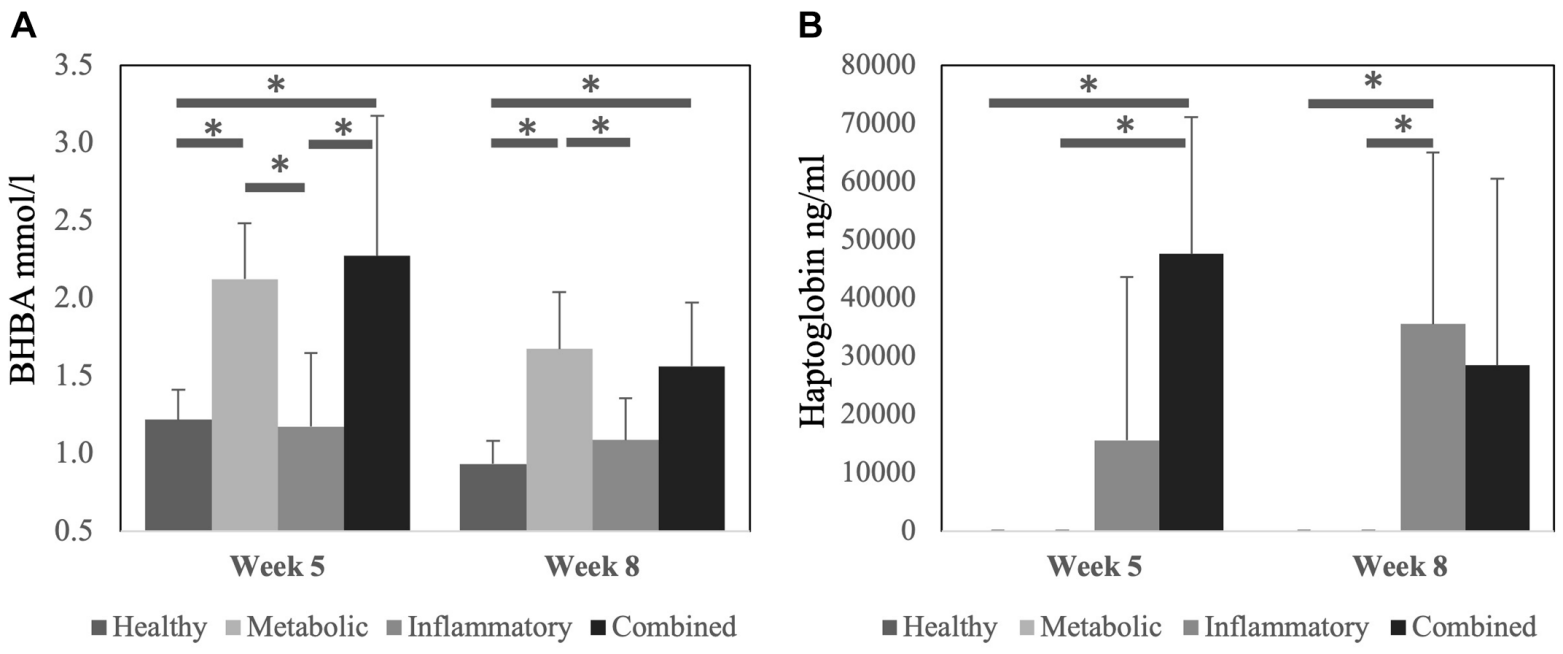
Concentration of **(A)** B-hydroxybutyrate acid (BHBA) and **(B)** haptoglobin in serum at 5 weeks and 8 weeks after calving according to the health status of the cows. Data is presented as mean ± SD. Bars with asterisk differ at: (*) *p* < 0.05.

Serum concentration of B-hydroxybutyrate acid (BHBA) was higher at week 5 postpartum in META and COMB cows compared to HEAL and INFL cows (*p* < 0.05) (Figure 1A). At week 8 postpartum, a similar pattern among groups was observed, except for the lack of statistical difference between COMB and INFL cows. Serum haptoglobin concentration was higher at 5 weeks postpartum in COMB compared to HEAL and META cows and at 8 weeks postpartum in INFL compared to HEAL and META cows (*p* < 0.05 for all comparisons) (Figure 1B). The parity of the cows was similar (*p* > 0.05) at the start of the experiment among the different health groups: HEAL: 2.3 ± 1.4, META: 2.0 ± 1.2, INFL: 2.0 ± 0.9 and COMB: 3.1 ± 1.6, respectively.

### 3.2 Body condition score and back fat thickness

**Figure 2:**
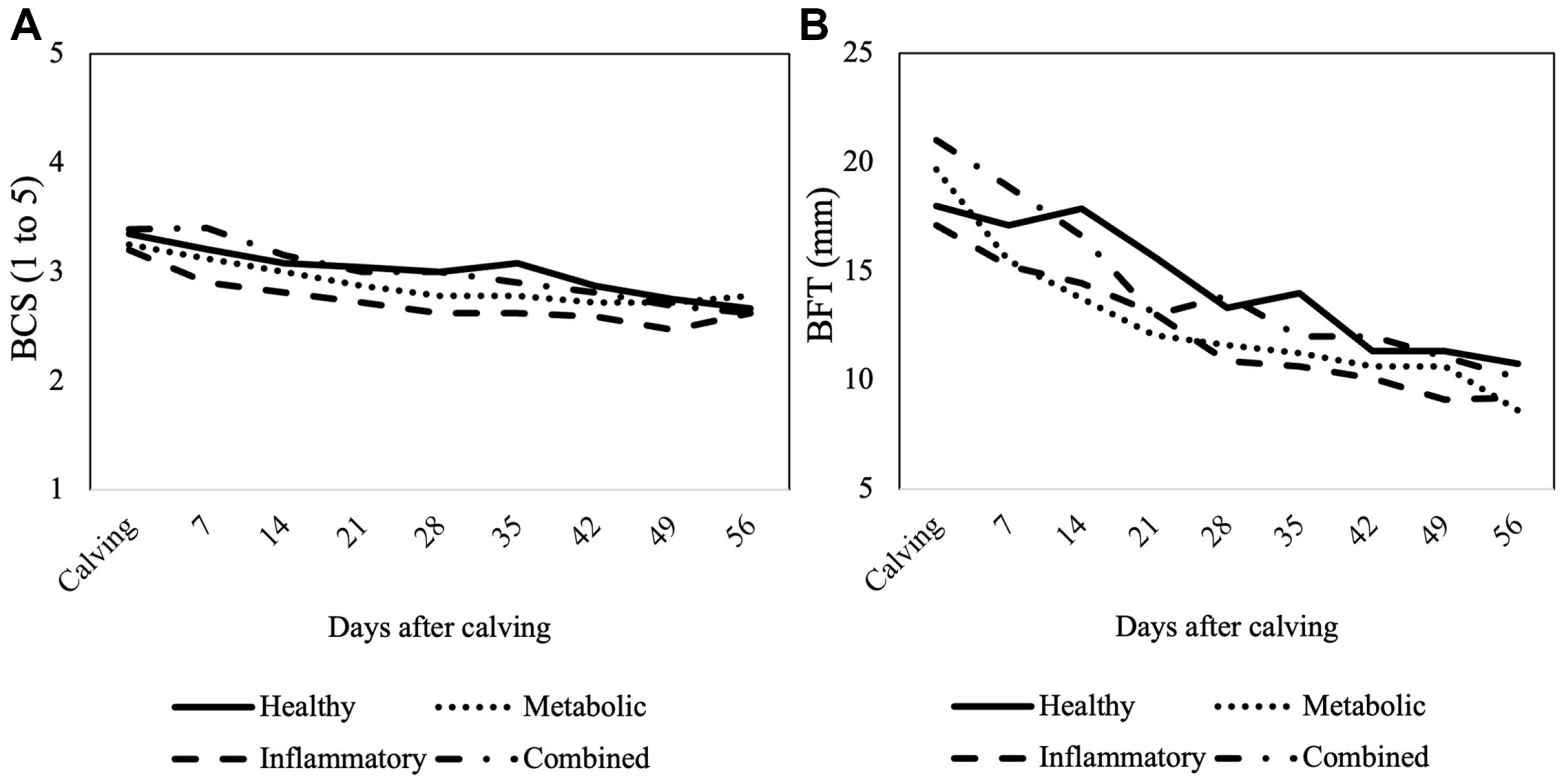
Mean values of **(A)** body condition score (BCS) and **(B)** back fat thickness (BFT) assessed from calving until week 8 postpartum according to the health status of the cows.

At the beginning of the study, BCS of cows was 3.2 ± 0.4 and BFT was 15.2 ± 4.3 mm, respectively. There was a significant decrease in BCS and BFT during the study period in all groups (*p* < 0.001 for both variables; Figure 2), but they were not associated with the health status of the cows (*p* = 0.525 for BCS and *p* = 0.847 for BFT). Also, no effect of the interaction group × week could be observed (*p* = 0.710 for BCS and *p* = 0.936 for BFT).

### 3.3 OPU outcome

**Figure 3:**
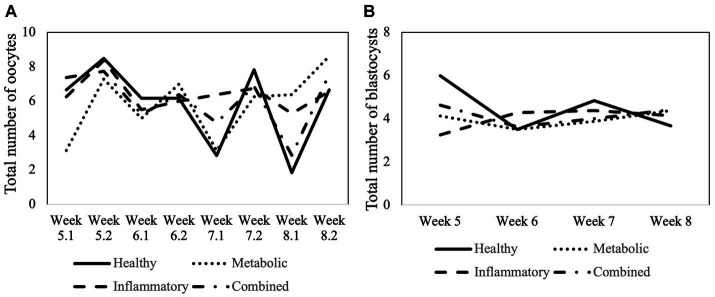
Total number of **(A)** collected oocytes and **(B)** *in vitro* produced embryos from week 5 to week 8 postpartum according to the health status of the cows. In case of the oocytes, the week number (5 to 8) is followed by 1 (first OPU during the specific week) or by 2 (second OPU during the specific week).

In total, 48, 63, 62, and 62 OPUs were performed on cows from HEAL, META, INFL, and COMB group, respectively. In one cow for each of the META, INFL, and COMB groups, one or two OPUs in the end were omitted as clinical signs of disease occurred and treatment was started. A total of 1,424 COCs were collected from 2,862 aspirated follicles across all groups (49.8% recovery rate). Neither of these parameters was influenced by the operator performing OPU (*p* > 0.05 in all cases). The number of aspirated follicles and retrieved oocytes for each group is presented in Table 1. The donor’s health status did not influence the number of oocytes collected per OPU or the recovery rate (*p* > 0.05 for both variables). Moreover, there was no time effect on the number of collected oocytes (Figure 3A) (*p* > 0.05).

**Table 1:** Number of aspirated follicles and recovered oocytes according to health status of the cows.

	HEAL	META	INFL	COMB	*p* value
OPU sessions	48	63	62	62	n/a
Follicles aspirated	566	748	758	790	n/a
Oocytes recovered	280	367	397	380	n/a
Oocytes per OPU	5.8	5.8	6.4	6.1	*p* > 0.05
Recovery rate (%)	49.5	49.1	52.4	48.1	*p* > 0.05

HEAL, healthy cows; META, cows with metabolic disease; INFL, cows with inflammatory disease; COMB, cows with combined metabolic and inflammatory diseases. Data on the average number of oocytes per OPU and recovery rate is presented as means.

### 3.4 Oocyte quality

Oocytes recovered by OPU (*n* = 1,424) were classified according to their quality (grade 1–4) as described in the 5th IETS Manual and their distribution into quality classes in relation to the health status of the cow was assessed. The number of high-quality oocytes (grade 1) was reduced in INFL and COMB cows compared to HEAL and META cows (*p* < 0.001; Table 2). Cows in the COMB group had more grade 2 oocytes compared to the other groups (*p* < 0.001; Table 2). The percentage of low-quality oocytes (grade 4) was increased in INFL cows compared to META cows (*p* < 0.001), but was similar to HEAL and COMB cows (*p* > 0.05; Table 2).

**Table 2:** Percentage of oocytes with quality (grade) 1 to 4 from 1,424 oocytes collected by OPU in accordance with the health status of the donor cow.

Oocyte quality grade	HEAL (%, *n*)	META (%,)	INFL (%, *n*)	COMB (%, *n*)	*p* value
1	39.3^a^ (*n* = 110)	39.5^a^ (*n* = 145)	24.9^b^ (*n* = 99)	23.4^b^ (*n* = 89)	*p* < 0.001
2	21.8^a^ (*n* = 61)	21.5^a^ (*n* = 79)	19.4^a^ (*n* = 77)	30.3^b^ (*n* = 115)	*p <* 0.001
3	22.9 (*n* = 64)	24.5 (*n* = 90)	27.5 (*n* = 109)	22.1 (*n* = 84)	*p* > 0.05
4	16.1^ab^ (*n* = 45)	14.4^a^ (*n* = 53)	28.2^b^ (*n* = 112)	24.2^ab^ (*n* = 92)	*p* < 0.001

HEAL, healthy cows; META, cows with metabolic disease; INFL, cows with inflammatory disease; COMB, cows with combined metabolic and inflammatory diseases. Group-related differences are flagged with different superscripts within the same row.

### 3.5 Embryonic development

Oocytes recovered by OPU from donors with different health status underwent maturation, fertilization and culture. Cleavage, blastocyst, and hatching rates were compared among groups. These results are summarized in Table 3. Cleavage rate was expressed as the percentage of cleaved embryos in relation to the total number of oocytes. Blastocyst rate was recorded as the percentage of embryos reaching the blastocyst stage in relation to the total number of oocytes placed in culture. Hatching rate was described as the percentage of hatched embryos in relation to the total number of blastocysts. Health status of the donor did not influence any of the abovementioned parameters (*p* > 0.05 in all cases; Table 3). Moreover, the number of produced blastocysts was not affected by the week postpartum or by the interaction between postpartum week and health group (Figure 3B) (*p* < 0.05).

**Table 3:** Percentage of *in vitro* embryonic development rates in accordance with the health status of the donor cow.

	HEAL (%, *n*)	META (%, *n*)	INFL (%, *n*)	COMB (%, *n*)	*p* value
Cleaved	83.4 (*n* = 136)	82.3 (*n* = 186)	84.6 (*n* = 170)	76.4 (*n* = 165)	*p* > 0.05
Blastocyst D7	44.8 (*n* = 73)	42.9 (*n* = 97)	43.3 (*n* = 87)	48.1 (*n* = 104)	*p* > 0.05
Blastocyst D8	58.9 (*n* = 96)	52.7 (*n* = 119)	54.2 (*n* = 109)	56.9 (*n* = 123)	*p* > 0.05
Blastocyst D9	66.3 (*n* = 108)	56.2 (*n* = 127)	59.7 (*n* = 120)	59.7 (*n* = 129)	*p* > 0.05
Hatched	75.9 (*n* = 82)	69.3 (*n* = 88)	64.2 (*n* = 77)	75.2 (*n* = 97)	*p* > 0.05

HEAL, healthy cows; META, cows with metabolic disease; INFL, cows with inflammatory disease; COMB, cows with combined metabolic and inflammatory diseases. D0: IVF day, D7: day 7 after IVF, D8: day 8 after IVF, D9: day 9 after IVF. According to the health group, 163 COCs in HEAL, 226 COCs in META, 201 COCs in INFL and 216 COCs in COMB, respectively, underwent IVF. Data are presented as means.

### 3.6 Embryo quality

As depicted in Table 4, most embryos reaching the blastocyst stage after fertilization and culture were categorized as quality 1 embryos, regardless of the health status of the donor. Less quality 1 embryos were observed in META and COMB cows compared to HEAL and INFL cows (*p* < 0.001 for both comparisons; Table 4). In addition, HEAL and COMB cows had the lowest and the highest percentage of quality grade 2 embryos, respectively (*p* = 0.003; Table 4). The percentage of quality grade 3 embryos was highest in META and COMB cows (*p* < 0.001), whereas the percentage of quality grade 4 embryos was similar across all groups (*p* > 0.05 in all cases; Table 4).

**Table 4:** Percentage of embryos (mean ± SD) with quality (grade) 1 to 4 after fertilization from donor cows with different health status.

Embryo quality grade	HEAL (%, *n*)	META (%, *n*)	INFL (%, *n*)	COMB (%, *n*)	*p* value
1	89.0^a^ (*n* = 73)	60.2^b^ (*n =* 53)	81.8^a^ (*n =* 63)	54.6^b^ (*n =* 53)	*p* < 0.001
2	7.3^a^ (*n =* 6)	22.7^b^ (*n =* 20)	16.9^b^ (*n =* 13)	28.9^c^ (*n =* 28)	*p =* 0.003
3	2.4^ab^ (*n =* 2)	13.6^bc^ (*n =* 12)	1.3^a^ (*n =* 1)	14.4^c^ (*n =* 14)	*p* < 0.001
4	1.2 (*n =* 1)	3.4 (*n =* 3)	–	2.1 (*n =* 2)	*p* > 0.05

HEAL, healthy cows; META, cows with metabolic disease; INFL, cows with inflammatory disease; COMB, cows with combined metabolic and inflammatory diseases. Data is presented as means. Group-related differences are flagged with different superscripts within one row (*p* < 0.05).

### 3.7 Morphokinetic parameters

Embryonic development was analyzed by TLM and the time of first cell divisions, duration of lag phase, start and the end of blastocyst expansion, and hatching were recorded (Table 5). There was no difference between healthy and diseased cows regarding the time required by presumptive zygotes to reach subsequent developmental stages (Table 5). First (t1) and second (t2) cleavage occurred later in embryos of INFL cows compared to embryos of COMB cows (*p* < 0.05; Table 5), but did not differ from HEAL cows (*p* > 0.05; Table 5). On the opposite, embryos of INFL cows were faster to complete hatching compared to those of COMB cows (*p* < 0.05; Table 5), but they did not differ from HEAL cows (*p* > 0.05; Table 5). The average time required for t3, tRCl, tSB, and tHB stages, as well as duration of lag phase, were similar among all groups for embryos which developed into blastocysts (*p* > 0.05; Table 5).

**Table 5:** Group-related time differences (hours) required by the embryos which developed into blastocysts to reach specific developmental stages.

	t1	t2	t3	dLP	tRCI	tSB	tHB	tHDB
HEAL	30.2 ± 3.8^ab^	37.4 ± 3.2^ab^	50.6 ± 4.5^ab^	44.4 ± 10.1	95.1 ± 11.6	163.7 ± 14.1	186.9 ± 10.6	192.0 ± 9.5^ab^
META	32.7 ± 4.2^b^	37.6 ± 4.4^ab^	50.1 ± 4.3^ab^	41.0 ± 6.9	91.0 ± 7.1	166.7 ± 19.7	183.2 ± 15.0	190.6 ± 14.1^ab^
INFL	33.6 ± 7.2^b^	40.6 ± 7.0^b^	52.1 ± 8.6^b^	43.2 ± 9.4	95.4 ± 13.0	158.0 ± 14.3	175.2 ± 9.8	179.5 ± 5.3^b^
COMB	30.0 ± 4.6^a^	37.4 ± 4.8^a^	47.7 ± 6.0^a^	41.4 ± 10.8	89.1 ± 11.0	158.0 ± 15.4	184.7 ± 13.7	193.1 ± 11.6^a^
*p* value	*p* = 0.008	*p* = 0.036	*p* = 0.025	*p* > 0.05	*p* > 0.05	*p* > 0.05	*p* > 0.05	*p* = 0.006

HEAL, healthy cows; META, cows with metabolic disease, INFL: cows with inflammatory disease, COMB: cows with combined metabolic and inflammatory diseases. t1: time of first cleavage, t2: time of second cleavage, t3: time of last cleavage before entering the lag phase, dLP: duration of the lag phase, tRCl: time of cleavage resumption after the lag phase, tSB: start of blastocyst expansion, tHB: start of hatching, tHDB: end of hatching. Data are presented as mean ± SD. Different superscripts within a column shown differences among groups (*p* < 0.05).

From the total number of 246 presumptive zygotes analyzed by TLM, direct cleavage was observed in 30 (12.2%) and reverse cleavage in 27 (11.0%) zygotes, respectively (Table 6). There was no difference in the percentage of zygotes with abnormal cleavage among groups. However, an analysis of variance based on logistic regression indicated that embryos of COMB cows were more likely to show direct cleavage compared to the HEAL cows (odds ratio = 5.38, *p* = 0.040). The incidence of direct cleavage was also more probable in embryos of the INFL group compared to HEAL; however, this trend failed to reach levels of statistical significance (odds ratio = 4.67, *p* = 0.057). The incidence of reverse cleavage was not affected by the health status in embryos that reached the blastocyst stage as well as for the total number of embryos (*p* > 0.05 in all cases). Simultaneous pairwise comparisons using Tukey’s HSD test did not indicate any difference between groups for both response variables (direct and reverse cleavage) (*p* > 0.05 in all cases).

**Table 6:** Absolute and relative counts of direct and reverse cleavage recorded in the embryos which developed to the blastocyst stage.

	HEAL	META	INFL	COMB
Direct cleavage				
No	28 (93%)	34 (83%)	33 (75%)	26 (72%)
Yes	2 (7%)	7 (17%)	11 (25%)	10 (28%)
Reverse cleavage				
No	26 (87%)	34 (83%)	33 (75%)	31 (86%)
Yes	4 (13%)	7 (17%)	11 (25%)	5 (14%)

HEAL: healthy cows, META: cows with metabolic disease, INFL: cows with inflammatory disease, COMB: cows with combined metabolic and inflammatory diseases.

## 4 Discussion

In the present study, we investigated whether the occurrence of subclinical disease in dairy cows during the transition period has an impact on the acquisition of developmental competence by the oocyte and the subsequent embryonic development. Collectively, our results show that cows diagnosed with subclinical metabolic and/or inflammatory disease during the first eight weeks after parturition have lower oocyte and embryo quality, respectively, but similar cleavage, blastocyst and hatching rates compared to healthy animals. The altered quality of oocytes and embryos might result from the exposure to a suboptimal follicular environment before acquisition of developmental competence. The present results also provide insights into morphokinetic characteristics of zygotes originating from subclinical diseased cows. The time required to reach specific developmental stages by zygotes from diseased cows was similar to those of healthy cows. However, a higher probability for abnormal cleavage as indicated by direct cleavage was observed in zygotes from diseased cows.

In accordance with a recent report where induced endometritis did not affect the number of collected oocytes (31), we did not detect any difference between healthy and diseased cows regarding number of oocytes recovered by OPU. Other authors showed that more oocytes could be recovered from cows with induced mastitis compared to healthy cows, but the number of viable oocytes and their quality were lower in the diseased group (51). It was hypothesized that the lack of feedback on the hypothalamus-pituitary axis due to reduced follicular fluid steroid concentrations is responsible for an increase in medium size follicles. Interestingly, induction of subclinical mastitis does not decrease follicular estradiol immediately but this effect occurs approximately two weeks later remarkably (52). Besides influencing estradiol concentration, mammary gland infections also trigger the production of pro-inflammatory cytokines which in turn influence GnRH and LH secretion and further interfere with follicular development (53, 54). However, hormonal treatments applied before OPU in the abovementioned study (51) should be also considered, as this has been shown to improve fertility in cows affected by mastitis (55). In our study, no hormonal treatment was applied to the cows before OPU, because the biweekly aspiration schedule has previously been associated with the highest number of harvestable follicles and more oocytes than other schedules (56).

A reduced oocyte quality was observed in cows suffering from inflammatory or combined metabolic and inflammatory disease compared to healthy and metabolic cows. Cows suffering from negative energy balance show time-dependent variations in serum concentrations of glucose, BHBA and urea during the first six to seven weeks after parturition, while the concentration of these metabolites in the follicular fluid of the dominant follicle follows the same pattern (9). However, oocyte quality is not impaired by increased BHBA concentrations or when oocytes are exposed for a short time to high NEFA concentration during postpartum period (57, 58). One protective barrier for the oocytes is represented by the cumulus cells which are able to partially counteract the negative effects of saturated NEFA (13); thus, cleavage rate is not affected (14). In the case of BHBA, which is one of the most abundant ketones in the bloodstream and concomitantly in follicular fluid of cows suffering from metabolic diseases, a dose-effect on blastocyst development rate, but not oocyte maturation or cleavage, was demonstrated (59). While BHBA and NEFA are products of adipose reserve mobilization, urea reaches the ovarian follicle through passive diffusion from the blood (60). Increased dry-matter intake leads to urea accumulation and it negatively affects embryonic development by reducing hatching rate as well as through increased apoptotic rate in the embryo (61). A similar effect has been observed for NEFA, as cell differentiation and hatching are impaired in developing embryos; remarkably, this adverse effect seems to be of maternal and not paternal origin (62). In order to detect alterations of the embryonic development occurring at a later stage, for example after transfer, we followed up on the produced embryos until day 9 after IVF and also checked their hatching capability. However, we were not able to find any effect of metabolic disease either on oocyte quality or on embryonic development. We suggest that subclinical ketosis does not influence the results of a commercial IVP program, likely due to intrafollicular BHBA, NEFA and urea concentrations below the toxicity level.

Interestingly, the negative effects of inflammatory diseases on bovine oocyte quality were not reflected in their developmental rates as cleavage, blastocyst or hatching rates were not affected by the presence of endometritis or mastitis. The same findings were previously reported by others (31, 63). Although induced mastitis with gram-negative bacteria can lead to a decrease in blastocyst rate (34), we were not able to detect any effect in cows with naturally occurring mastitis in our study. It is worth mentioning that cows included in our study suffered from subclinical mastitis, whereas the intramammary injection of gram-negative bacteria in the abovementioned study initiated strong clinical signs of disease, both locally and systemically. However, cows receiving an intramammary injection of gram-positive bacteria in the same study did not develop clinical signs of disease and the IVP results were also not affected (34). The hatching rate was also similar among groups, which is in accordance with the findings of a previous study (64). These results suggest a clear influence of the disease severity on IVP outcome. Nevertheless, long-term effects of inflammatory diseases present at the time of oocyte collection on the capability of the embryo to create a pregnancy and be carried to term should be further investigated. Inflammation products as interleukins and cytokines can lead to embryonic mortality in dairy cattle by disrupting the reproductive axis at various points including the oocyte and the embryo (65).

Our results showed a significant decrease in quality 1 embryos in cows with either metabolic or combined disease, but not inflammatory disease, compared to healthy cows. This suggests a carryover effect on the oocyte of metabolites released into follicular fluid in response to negative energy balance. Although bovine embryos exposed to increased concentrations of BHBA in the maternal environment can exhibit mitochondrial imbalances, slowed growth, and activation of protective mechanisms such as autophagy, no correlation has been observed between BHBA concentrations and embryo quality after flushing (21). In comparison to metabolic disease, the presence of inflammatory disease alone did not affect embryo quality. This aspect is intriguing, as significantly less high-quality oocytes were collected from cows with inflammatory disease. The usage of conditioned culture media previously exposed to an inflamed endometrium was shown to slightly reduce embryo quality by decreasing the cell number and increasing the inner cell mass:trophectoderm ratio (64). However, the developmental competence of the oocytes recovered from cows with inflammatory disease seems not to be affected, as they resulted into good quality embryos once removed from the inflammatory environment. Chronic inflammation can disrupt the delicate balance of growth factors and signaling molecules necessary for optimal embryonic development. The release of pro-inflammatory cytokines during inflammation interferes with the proper development of the embryo, leading to reduced embryo quality and lower pregnancy rates (27, 28). Nevertheless, a further decrease in embryo quality might occur at a later time, as inflammation exerts carryover effects for up to 4 months on reproductive function (66). Notably, inflammatory mediators can act directly on reproductive organs, but can also affect the brain and its related functions like LH secretion (67). Increased nutritional requirements during the transition period might decrease the concentrations of glucose, insulin, and IGF-1 and therefore compromise follicle growth and steroidogenesis (68). Therefore we cannot exclude effects on preantral follicle population which only become visible later during lactation, while in our study IVP was performed during the second month after calving.

The evaluation of embryo quality based solely on morphological criteria is subjective and therefore considered nowadays inadequate (37, 38). The analysis of embryo morphokinetics can help to select embryos with high implantation potential based on the time of first cleavage (36, 39). The average time required by the embryos in our study to reach specific developmental stages was similar between diseased and healthy cows, with just some differences occurring among diseased groups. In addition, we could not see any difference in the time required for the first or second cleavage between viable and non-viable embryos among all groups, as previously described (44). However, the embryos in the latter study required less time to undergo first cleavage than those in our study (25 vs. 30 h), which might be attributed to the breed (Japanese Black) or to the presence of serum in the culture media (69). Comparatively, similar morphokinetic patterns like in our study were described in other recent studies using slaughterhouse-derived oocytes (70, 71). Moreover, in one of these studies, the authors determined a cut-off point in time (32 h and 22 min) to differentiate between fast- and slow-cleaving embryos as a predictor for blastocyst development (70). This is different than the typical way to discriminate between fast and slow embryos based on the average time required for the first division by zygotes that develop to a blastocyst (41, 72). Interestingly, the cut-off point in time for first cleavage was met by all groups in our study, thus, the presence of subclinical disease seems not to affect the timing of divisions during embryo development.

Recently, it has been shown that multi pronuclei and migration error of pronuclei are the primary reasons for direct cleavage in cattle (73). Using TLM we were able to observe a higher probability of direct cleavage in zygotes of cows with combined and inflammatory disease. This might be due to the negative effects of intrafollicular inflammatory agents on oocyte spindle assembly, considering that abnormalities in oocyte microtubule positioning lead to failure in pronuclei apposition (73). As direct cleavage was previously associated with embryonic arrest in cattle (41, 74), it would be interesting to analyze the pregnancy rate after transfer of embryos from diseased cows produced in this study. In human medicine, direct cleavage embryos are not recommended for day 3 transfers, but can be transferred if they reach blastocyst stage in culture (75). Reverse cleavage has also been reported in bovine embryos in recent studies with an incidence of 11%–17% and its occurrence prevented the zygotes from reaching the blastocyst stage (41, 71). The occurrence of reverse cleavage is mainly attributed to the male factor, as semen characteristics at fertilization are strongly correlated to abnormal patterns of cell divison (76). This might also explain why we could not observe any difference in the incidence of reverse cleavage in this study, as only semen from one bull and from the same batch was used for IVF. However, our observations showed that zygotes with reverse cleavage can still reach the blastocyst stage, and therefore should not be removed from culture at early stages.

In conclusion, the presence of subclinical metabolic and/or inflammatory disease in dairy cows during transition period reduces oocyte and embryo quality without affecting their developmental potential. Moreover, morphokinetic parameters of zygotes originating from diseased cows were found similar to those of healthy cows. A higher incidence of abnormal cleavage was observed in embryos from diseased cows but these could still reach blastocyst stage. Future studies should determine whether the presence of subclinical disease can affect oocytes and/or embryos at a molecular level with detrimental effects on pregnancy rates after transfer.

## Data availability statement

The raw data supporting the conclusions of this article will be made available by the authors, without undue reservation.

## Ethics statement

The animal study was approved by Committee on Animal Experimentation of the Cantonal Veterinary Office Zurich (license no. 131/2018). The study was conducted in accordance with the local legislation and institutional requirements.

## Author contributions

IS: Formal analysis, Investigation, Writing – original draft, Writing – review & editing, Data curation. AG-G: Conceptualization, Investigation, Methodology, Supervision, Writing – review & editing. CH: Investigation, Methodology, Writing – review & editing. II: Investigation, Methodology, Writing – review & editing. MT: Investigation, Writing – review & editing. MM: Writing – review & editing, Investigation. FM: Writing – review & editing, Methodology. EM: Formal analysis, Writing – review & editing, Methodology, Software. HB: Supervision, Writing – review & editing, Conceptualization, Project administration, Resources. DS: Formal analysis, Investigation, Supervision, Writing – original draft, Writing – review & editing.
